# 3D-Imaging of Whole Neuronal and Vascular Networks of the Human Dental Pulp via CLARITY and Light Sheet Microscopy

**DOI:** 10.1038/s41598-019-47221-5

**Published:** 2019-07-26

**Authors:** Cristiane Miranda França, Rachelle Riggers, John L. Muschler, Matthias Widbiller, Peter Manning Lococo, Anibal Diogenes, Luiz Eduardo Bertassoni

**Affiliations:** 1 Division of Biomaterials and Biomechanics, Department of Restorative Dentistry, School of Dentistry, Oregon Health & Science University, Portland, OR USA; 2 Biomedical Engineering Department, Knight Cancer Institute, OHSU Center for Spatial Systems Biomedicine, Oregon Health & Science University, Portland, OR USA; 3 Department of Conservative Dentistry and Periodontology, University Hospital Regensburg, Regensburg, Germany; 4 Department of Endodontics, University of Texas Health Science Center at San Antonio, San Antonio, Texas USA; 5 Center for Regenerative Medicine, School of Medicine, Oregon Health & Science University, Portland, OR USA; 6 Department of Biomedical Engineering, School of Medicine, Oregon Health & Science University, Portland, OR USA; 7 Cancer Early Detection Advanced Research Center (CEDAR), Knight Cancer Institute, Portland, OR USA

**Keywords:** Dental pulp, Dental pulp, 3-D reconstruction, 3-D reconstruction

## Abstract

Direct visualization of the spatial relationships of the dental pulp tissue at the whole-organ has remained challenging. CLARITY (Clear Lipid-exchanged Acrylamide Tissue hYdrogel) is a tissue clearing method that has enabled successful 3-dimensional (3D) imaging of intact tissues with high-resolution and preserved anatomic structures. We used CLARITY to study the whole human dental pulp with emphasis on the neurovascular components. Dental pulps from sound teeth were CLARITY-cleared, immunostained for PGP9.5 and CD31, as markers for peripheral neurons and blood vessels, respectively, and imaged with light sheet microscopy. Visualization of the whole dental pulp innervation and vasculature was achieved. Innervation comprised 40% of the dental pulp volume and the vasculature another 40%. Marked innervation morphological differences between uni- and multiradicular teeth were found, also distinct neurovascular interplays. Quantification of the neural and vascular structures distribution, diameter and area showed that blood vessels in the capillary size range was twice as high as that of nerve fibers. In conclusion whole CLARITY-cleared dental pulp samples revealed 3D-morphological neurovascular interactions that could not be visualized with standard microscopy. This represents an outstanding tool to study the molecular and structural intricacies of whole dental tissues in the context of disease and treatment methods.

## Introduction

The dental pulp is a highly innervated and vascularized soft tissue that is responsible for most of the biological functions in the tooth. Odontoblast cells in the pulp form an immediate interface with dentin, the most abundant mineralized tissue in the tooth, and continuously secrete dentinal tissue over the life span of the tooth. Trigeminal ganglion neurons, which innervate the pulp, participate actively in the transmission of dental pain by conducting sensory impulses from pulp to the central nervous system^1,2^, and there is sufficient evidence that neural cells also contribute to the overall homeostasis of the pulp tissue in a paracrine and juxtacrine manner^3,4^. Blood capillaries, on the other hand, allow for cell nutrition, oxygenation, and removal of metabolic products^5^, and together with the neural component, orchestrates most of the physiologic and pathologic responses of the tooth^3,6,7^. Therefore, a thorough understanding of the multiscale structure and function relationships of constituents of the dental pulp is imperative for an improved understanding the physiologic and disease conditions affecting the tooth, as well as the development of new strategies for vital pulp therapy.

Significant efforts have been expended toward characterizing the structural organization of the innervation and vasculature in the dental pulp. Early resin cast^8,9^ and Indian ink^10^ studies have provided substantial evidence of the vascular network architecture in dental pulp. Nevertheless, these studies enabled little molecular characterization of the tissues in question, or insight into how vessels may interface with the innervation, because the casting and the ink allows for reconstructions of hollow resin-infiltrated blood vessels only. More recently, studies combining light and electron microscopy^11,12^ have provided more details of the interface between vessels and nerves in the dental pulp with a greater level of structural precision. However, the current knowledge of the dental pulp vasculature and innervation is mostly limited to 2D-images, or partial 3D-reconstructions^13,14^ of small sections of the tissue which only allow analysis of short and random segments. Standard histology and immunohistochemistry typically require tissue sectioning down to thin layers, which may damage or distort the tissue, and lose much of the 3D structural information at the whole- organ level^13–17^. Moreover, the serial sections may present considerable variability in staining and may require long periods of time to reconstruct the whole tissue. Confocal microscopy, on the other hand, enables imaging of relatively thicker samples (100 to 200 µm)^18^, however, rendering of 3D structural information for an entire organ system, such as the whole tooth, is virtually impossible due to physical limitations of the optics/objectives (e.g., numerical aperture/working distance relationship and resolution, refractive index matching, light scattering in general, etc.). As a consequence, our understanding of the structural, functional and molecular relationships of the whole dental pulp, in 3D and in relation to their position in the tooth have remained limited thus far, particularly with respect to the innervation and vasculature.

CLARITY, or Clear Lipid-exchanged Acrylamide Tissue hydrogel, has been a successful tool to allow for optical access into an intact tissue at the whole- organ level, down a single capillary or neuron^17^. Briefly, lipid bilayers in cells that form tissues in the body have long been implicated in light scattering^19^, which limits the capability of most light microscopy techniques in sampling thick tissues^17^. The CLARITY method functions by transforming cell-rich tissues into a lipid-free tissue-polymer hybrid. First, the proteins, nucleic acids and macromolecules are stabilized with formaldehyde and hydrogel monomers (acrylamide and bisacrylamide). Next, after the tissue and hydrogel are polymerized, lipids are removed using various detergents, such as sodium dodecyl sulfate (SDS), leaving the proteins in their original position, thus resulting in a completely transparent tissue that has virtually intact structural integrity^17,20^. CLARITY-cleared tissues can be immunolabelled with a wide range of markers, and imaged using standard microscopy methods or using light sheet microscopy which allows for faster processing times and increased imaging depth^17,21–23^. While extensive work with CLARITY and other similar clearing techniques has been done in brain tissue, ovary^24^, lung^23^, bone^25^, pancreatic tissues^26^, and some aspects of murine teeth^27^, these clearing techniques have not been utilized to study the human dental pulp.

Here we demonstrate the first application of CLARITY to the analysis human teeth, with a special emphasis in the dental pulp innervation and vasculature. We provide evidence and quantitative characterization of the complex interconnectivity of the dental innervation and vasculature, starting from the tooth apex all the way to the coronal pulp-dentin interface, with direct manipulation over XYZ visualization modes of these two constituents at micrometer scale resolution. We argue that this work may pave the way for greater insight into the structure and function relationships of the vascular and neural components in the dental pulp, and how they may respond to endodontic therapies and regenerative interventions in the future.

## Results and Discussion

### CLARITY preserves dental pulp morphology and enables visualization of the whole tissue

**Figure 1:**
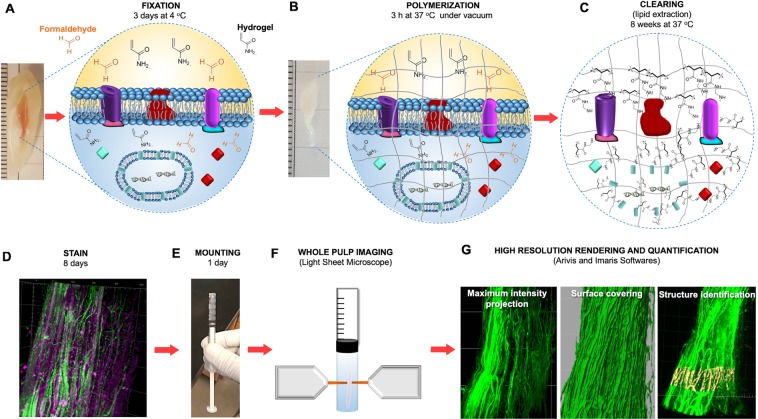
Schematic of the optimized CLARITY process to clear human dental pulp. Freshly extracted dental pulp samples were embedded in formaldehyde, acrylamide and bisacrylamide monomers. (**A**) Amine groups from proteins were covalently linked to monomers and polymerized into a hydrogel. (**B**) Lipids are passively removed with a SDS buffer. (**C**) The tissue-polymer hybrids are stained with CD31 and PGP9.5 antibodies (**D**), mounted in an agarose plug (**E**), imaged with a light sheet microscope (**F**) and images were rendered and quantified using Arivis and Imaris softwares (**G**) showing several possibilities of image visualization, as maximum intensity projection surface covering and neural bundles identification.

Adult canine and premolar teeth extracted for orthodontic reasons had the dental pulp carefully removed and immersed in a 4% formaldehyde, acrylamide and bisacrylamide solution for 72 hours at 4 °C (Fig. 1A). The hydrogel pre-polymer was then thermally polymerized by incubation at 37 °C for 3 h inside a vacuum chamber filled with nitrogen gas (Fig. 1B) forming a meshwork of hydrogel covalently linked to native proteins, small molecules and nucleic acids. Subsequently the dental pulp/hydrogel hybrids were transferred into conic tubes and immersed in a clearing solution of SDS and boric acid for 6–8 weeks at 37 °C in a passive method to remove the lipid bilayers resulting in a semi-transparent tissue/hydrogel hybrid with preserved structural and molecular information (Fig. 1C). This hybrid tissue was stained for PGP9.5, a neuron specific marker, and for CD31, an integral membrane glycoprotein which is present in high levels on early and mature endothelial cells, especially at junctions between adjacent cells (Fig. 1D). The immunostained hybrid-tissues were then imaged using light sheet and confocal microscopy (Fig. 1E–G)^21,28–31^.

**Figure 2:**
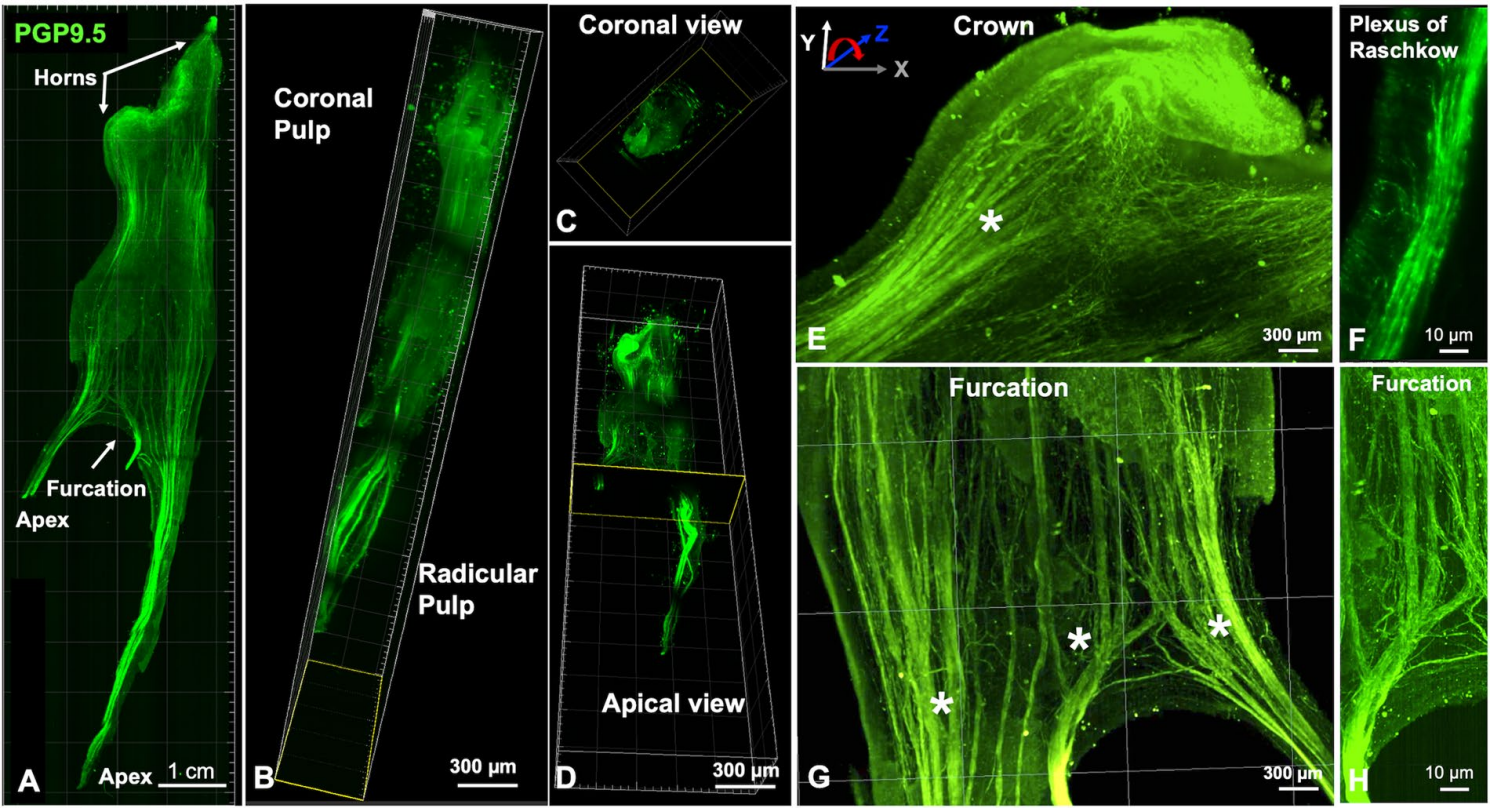
Whole innervation of a human premolar from a mesio-distal view (**A**), a palatal-vestibular perspective (**B**), an occlusal (**C**) and an apical-coronal view (**D**). In the high-magnification images from the crown it is possible to observe several nerve bundles that run straightforward in the coronal direction (**E**) splitting (*) down to the level of a single nerve fiber from the plexus of Raschkow directing towards the dentin site. (**F**) In the furcation level it can be observed thick bundles that come from the root and start to split (*) determining the site-specific innervation of the pulp chamber (**G**,**H**) (Immunofluorescence, PGP9.5 - green, CD31 – red).

In order to image the entire tooth structure, images were obtained as separate sequential portions, or tiles, and were then rendered separately. After rendering, the tiles were stitched forming the complete image in 3D that could be analyzed in different ways (Fig. 1G) as maximum intensity projection, surface covering, structure identification and quantification. For example, the premolar pulp had 16 mm in length and could be imaged in 4 different tiles that were stacked to form the whole 3D-image (Fig. 2A,B,E) (Supplementary Movie 1). Imaging of such large sample was possible by use of light sheet microscopy technique, which uses a pair of illumination objectives to provide a planar sheet of light that irradiates a thin section of the specimen at each slice. The detection objective is located at an orthogonal axis related to the illumination, maximizing detection efficiency and minimizing fluorescence from out-of-focus features and bleaching^32^, which would be expected with traditional confocal and fluorescence microscopy.

### Dental pulp innervation

**Figure 3:**
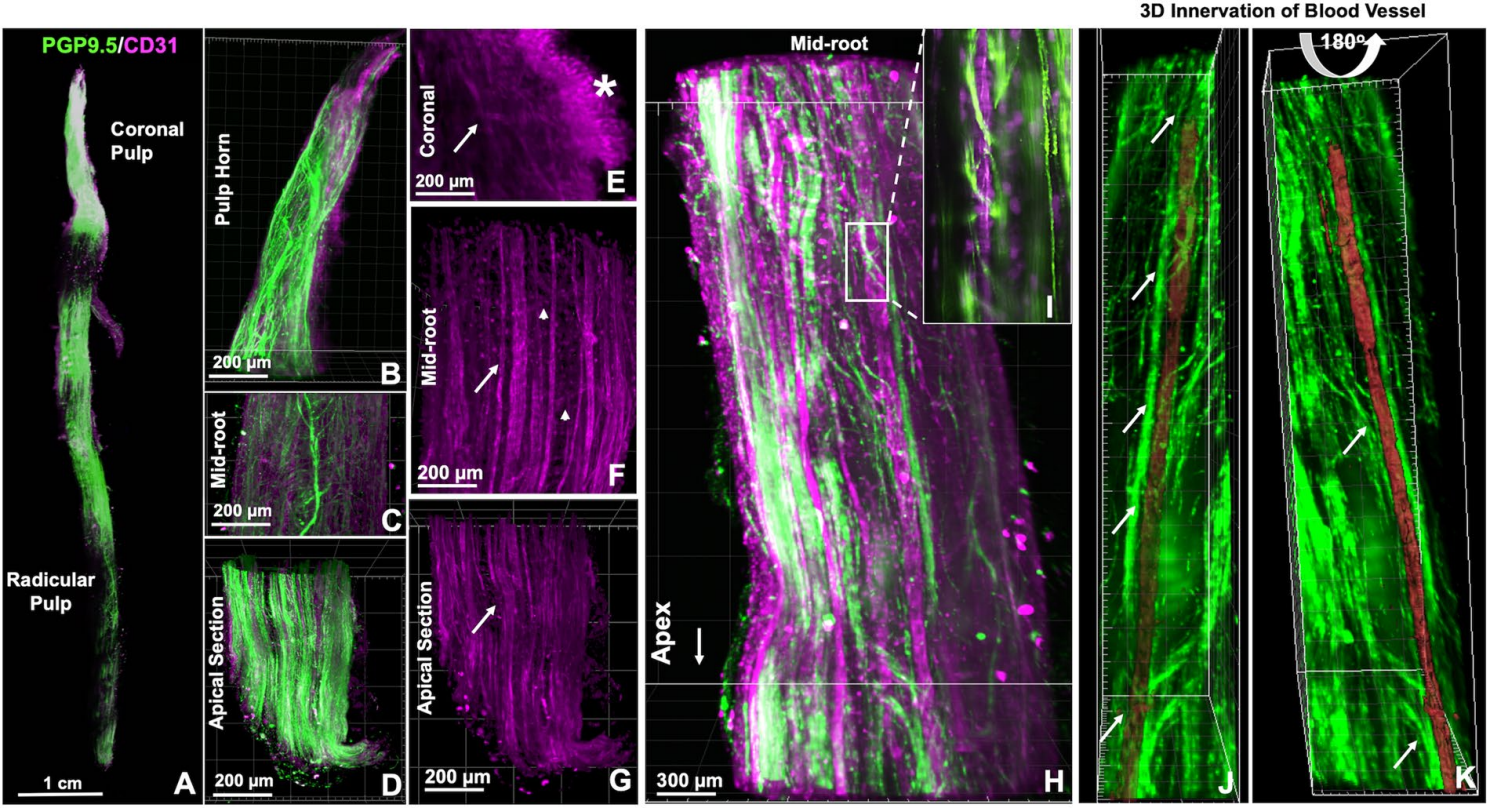
Dental pulp from human canine tooth showing the neurovascular structures after CLARITY processing and imaging.(**A**) 3D reconstructions show predominantly nerve bundles and vessels running parallel to the long axis of the tooth (**D,G**), in the mid-root region there is a splitting of the neural structures whereas core vessels present a straight course (**C,F**) At the coronal area (**B,E**) nerves and vessels (arrow) show marked ramification, mostly in the sub-odontoblastic region (*). In the mid-root area (**H**) complex neurovascular interplay are observed including transversal connections between vessels (arrow heads) (**H**) and the inset illustrates an area of interest in one of the core vessels, which is surrounded by spiraling nerve fibers. (**I**) A region of interest was selected (**I,K**) and the innervation from different neural bundles was identified (arrows). The vessel of interest was highlighted, and after deactivation of the red channel, the vessel was analyzed in different planes of Z-stack rendering including a 180° rotation to show the innervation (arrows) from different 3D-perspectives – front and back. (Immunofluorescence, PGP 9.5 - green, CD31 – red).

The images were rotated such that the 3D characteristics of the innervation in the tooth apex, furcation area, coronal pulp and pulp horns could be investigated either separately or as a whole. At the foramina level multiple neural bundles run parallel to the long axis of the root (Figs 2A–G and 3A,D) (Supplementary Fig. 1I–L). The 3D rotation of the uniradicular teeth enabled the visualization of the apical and mid-root outer neural structures, close to the dentin walls (Fig. 3B, Supplementary Fig. 1K,L). The peripheral bundles split thin nerve fibers that have a diagonal upward course, reaching out to other distant peripheral bundles located as far as 2 or 3 mm from one another, forming a transversal network in the boundaries of the root canal which connects one side of the tooth to another (Fig. 3C,H and Supplementary Fig. 1K,L, Supplementary Movie 2). Nerve bundles in the core of the root canal present pronounced splitting directed transversally to the tooth axis or in 45°, following either a straight line toward the dental pulp periphery, or a wavy course inside the dental pulp core, or even loop backwards in a reverse direction (Fig. 3C,H, and Supplementary Fig. 1G,H, Movie 3). Instead, in the multiradicular teeth almost no splitting is seen in the apical and middle thirds of the tooth, corresponding to the length of the root, also a marked branching and splitting is present near the floor of the pulp chamber, (Fig. 2A,E,G,H). This finding characterizes a distinctive pattern of splitting for peripheral and core nerve bundles between uni- and multiradicular teeth, which is only possible to track if a root section of at least 5 mm in length is imaged, analyzed with 3D rotations and further examined layer by layer with XY and XZ slices, as enabled by CLARITY and light sheet microscopy, but not possible with standard microscopy techniques.

The 3D optical access to the images enabled us to track the course of a nerve bundle of interest (Supplementary Movie 4), and also to investigate several points of interplay with blood vessels (Supplementary Movie 5). Of note, in the premolars it is possible to precisely determine that the nerve bundles that transverse the root canal to reach the coronal pulp give off only a few collateral branches that do not cross from one side of the tooth to the other (Fig. 2A) (Supplementary Movie 1). Differently from the uniradicular teeth, these axons respect a clear boundary for the innervation where palatal root innervates the palatal side of the tooth, the vestibular root splits into the vestibular area of the tooth, and the nerve bundle that enters into the furcation innervates the central part of the tooth. The specific axon extension, guidance and branching to appropriate targets is likely to be modulated by molecular signaling given by the extracellular matrix^33,34^ and growth factors^35^, and are far from being fully understood, so the use of cleared whole teeth can shed light on the establishment of pulp innervation in healthy conditions, in the presence of various challenges, or after regenerative procedures.

At the coronal pulp and pulp horns the nerve bundles undergo ramification with multiple branching and several terminal endings close to the dental pulp periphery (Fig. 2E) (Supplementary Fig. 1). Given that the gradual transition from thicker nerve bundles to the narrower nerve fibers in the plexus of Raschkow occurs over the extent of a several micrometers, and at different locations in the whole tooth structure, the analysis of the cleared whole tooth enabled the imaging and mapping of the transition of the dental pulp innervation from the thick apical nerve bundles up into the single nerve fibers in the plexus of Raschkow (Fig. 2F), also allowing for the determination of the exact point where fibers split, branch and interface with blood vessels (Fig. 2C,D). Additionally, this detailed optical access is possible in the different areas of the dental pulp and with a range of magnification from centimeters to 1 micron (Fig. 2) (Supplementary Movies 1 and 6).

Of note, even though we observed neural structures with splitting patterns that have been described before in the literature^12,36–38^, in this study the fresh pulps were pulled out from the dentin and then fixed, giving rise to the possibility of distortions in the more delicate areas of the tissue. Especially for molars with multiple roots where it is quite challenging to preserve the dental pulp structure while pulling out the soft tissue from dentin which can be a potential limitation of this technique. For future studies, it would be beneficial to preserve the structural integrity of the neural network of the dentin-pulp complex by demineralizing, processing and imaging pulp and dentin as a whole.

### Dental pulp vasculature

Similar to the innervation, the main blood vessels in the dental pulp pass through the apical foramen, run towards the coronal part of the root canal (Fig. 3C–F) giving rise to a rich capillary network in the subodontoblastic area (Supplementary Fig. 1). It has been described that the main arterioles that enter the apical foramen have approximately 10–50 μm in diameter and are preferentially located in the central portion of the root canal^39^. Even though the average diameter of blood vessels decreases in the coronal area up to the capillary level, we observed that the coronal dental pulp presents core vessels with diameters of 14–10 microns, (Supplementary Fig. 1), which are compatible with the descriptions of the central veins, that perform the venous drainage towards the apical foramen^10^ (Fig. 3).

In the whole extension of the dental pulp, transversal connections between core vessels can be identified, (Fig. 3F, Supplementary Movie 7), resembling the characteristics of arteriovenous connections, important regulators of the dental pulp blood flow^8–12,40^. However, further studies including ultrastructural arteriole and vein characterization would be necessary to confirm this finding.

It is known from the literature that beneath the dentin layer numerous arterioles branch out perpendicular or obliquely from core vessels forming the subodontoblastic capillary plexus^8,9,41^. At this point, terminal capillaries show a diameter of 6–10 μm, and then progressively convert into venules that will anastomose with larger vessels to conduct the blood flow out of the tooth via the apical foramen^9,12^. In our samples, the dental pulp sub-odontoblastic vascular plexus (Fig. 3E) was comprised of such a dense vascular network with an inherent background that made it challenging to image in low-magnification (Supplementary Fig. 2). Additionally it is known that other cells as monocytes, neutrophils, leukocytes and some dental pulp cells can express CD31, so to exclude non-endothelial layer, future studies could consider to label the tissue with a second marker for blood vessels.

### Innervation of the dental pulp vasculature

**Figure 4:**
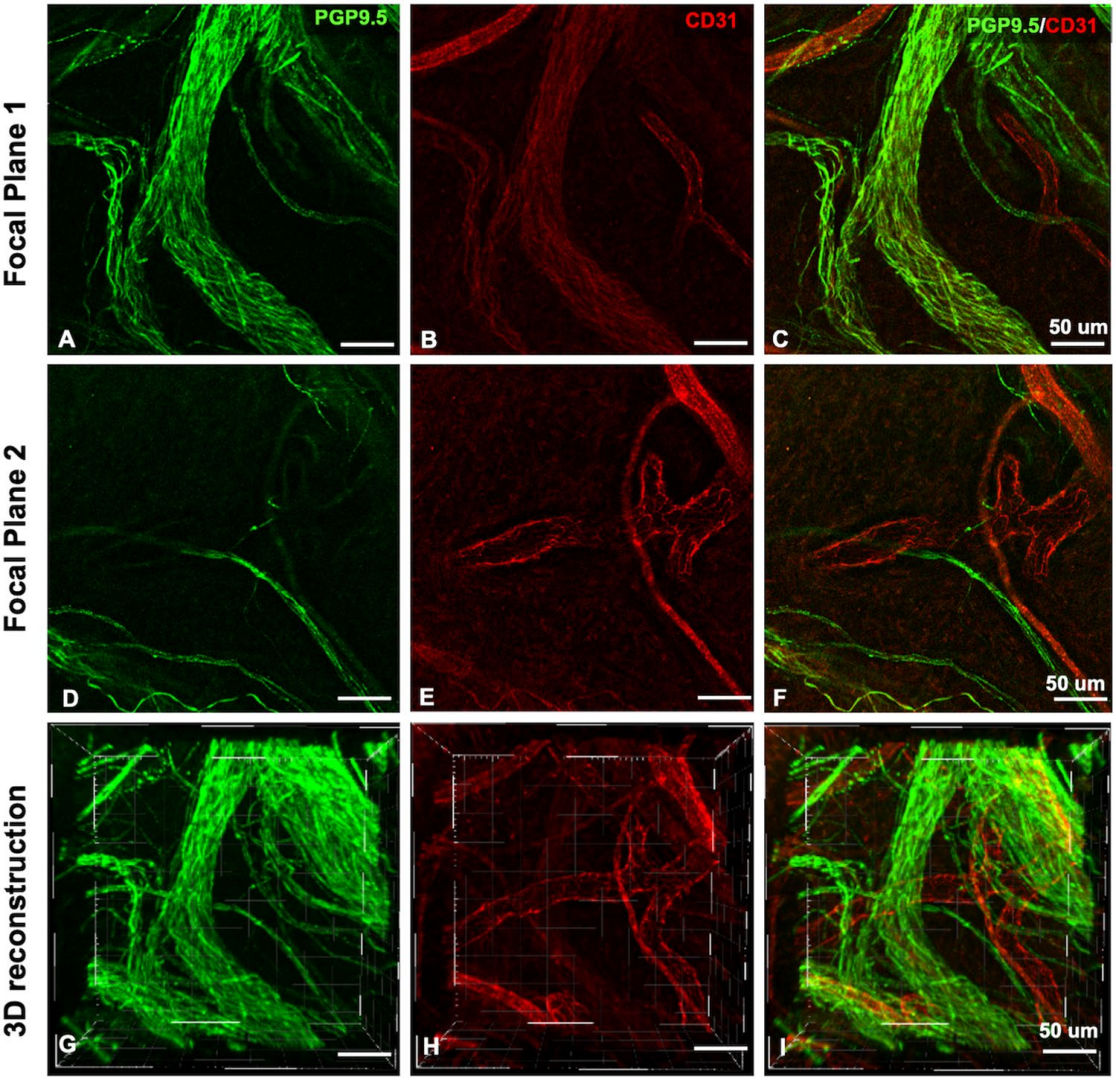
Neurovascular bundles in 3D at the furcation level showing the blood vessels ranging from 4 to 30 µm. (**A–C**) The focal planes 1 and 2 have a difference of 100 µm in the Z-distance, and illustrate the complexity of dental pulp neural and vascular interplay. Some blood vessels were embedded into the neural structures and others were either innervated by single nerve fibers (**D–F**) or did not show innervation at the level of the imaged site. (**F**) (Immunofluorescence, PGP9.5 - green, CD31 – red, confocal 20x objective).

The innervation of dental pulp blood vessels has been object of much investigation^11,13–16,39–41^, however, considerable information on the neurovasculature interplay is lost when the tissue is sectioned^17,40^. The cleared whole dental pulp offers unique information on the 3D configuration of the perivascular neural plexuses and the interplay between the neural structures and the vasculature. Most of the dental pulp neurovascular bundles were arranged in the three different patterns that have been classically stated in the literature: (i) thick nerve bundles alongside the blood vessel extension, (ii) thin spiral innervation from splits of nearby nerve bundles and (iii) neural bundles that completely surrounded blood vessels (Fig. 4, Supplementary Figs 2B,C, 3, and Movie 8). Among the new evidence that our study brings, a few key aspects are noteworthy, for instance: (i) all of the abovementioned patterns of innervation can observed in different regions of a same vessel (Fig. 3H,I); (ii) a single blood vessel can be innervated by nerve fibers that split from different bundles in a sequence of innervated areas intercalated with non-innervated areas (Fig. 3H–J); (iii) virtually all vessels from the dental pulp are innervated at some level during their course throughout the pulp (Supplementary Movie 7), and (iv) the splitting of nerve bundles into adjacent vessels creates a tortuous complex neurovascular network inside the dental pulp that has been poorly characterized from a 3D whole-tooth perspective so far (Fig. 4C,F,I) (Supplementary Movies 1, 5 and 6).

Our finding that vascular-related nerve fibers presented either as single fibers or as prominent perivascular plexus corroborate previous studies that investigated the relationship of nerves and vessels in the dental pulp using immunohistochemistry of histological sections^37,40^. These studies focused on the analyzes of the central portion of the pulp due to the technical challenges of making histological sections, staining, imaging and reconstructing areas such as the furcation. A remarkable advantage of using CLARITY to study the dental pulp is that it provides optical access to the totality of the structures of pulp in the whole tooth, including in the more challenging areas in a way that the data can be quantified more precisely than using histological images.

### Quantification of neural and vascular components

**Figure 5:**
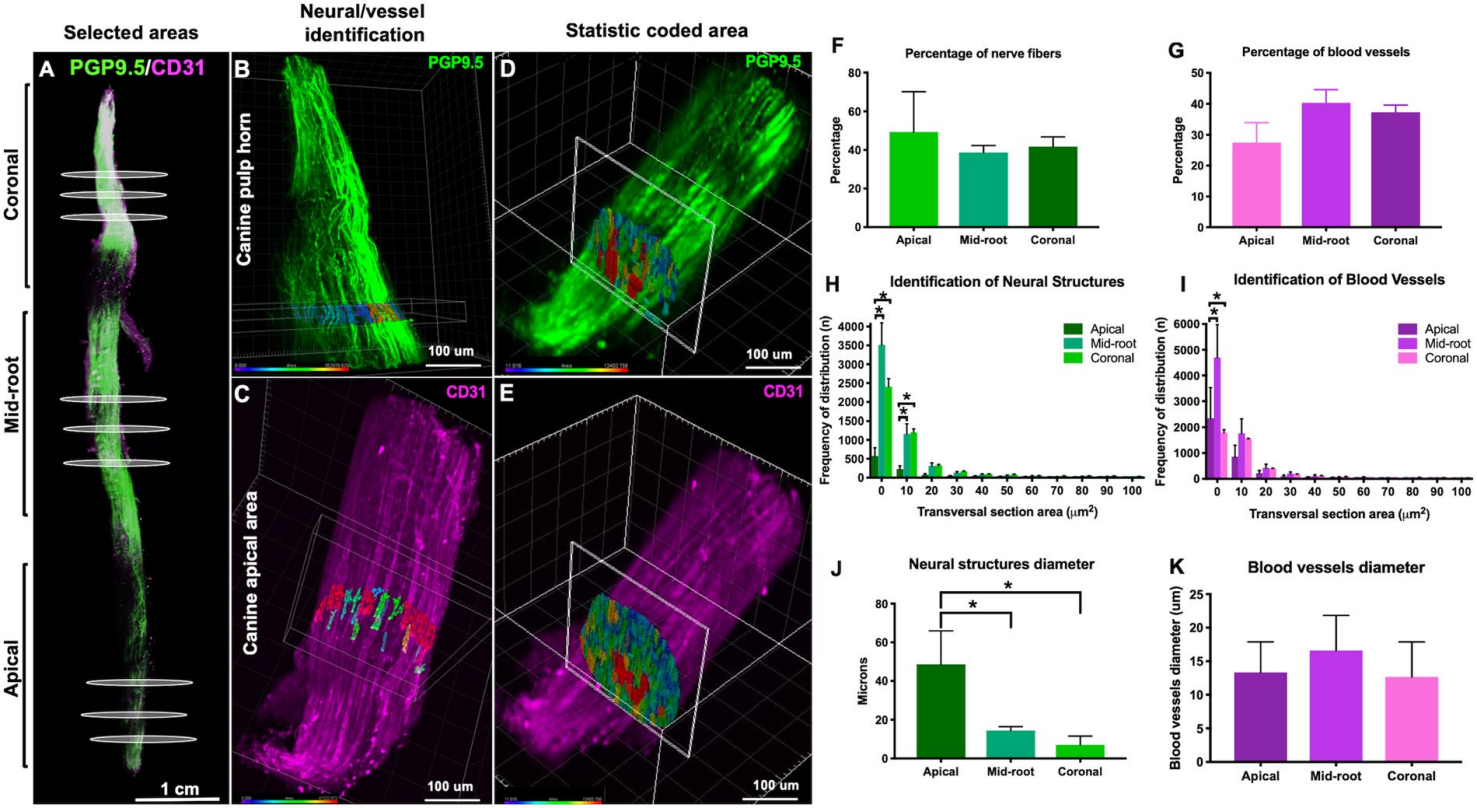
Selection of area of the whole tooth. (**A**) Areas occupied by nerves (**B**) and blood vessels (**C**) in a segment of 10 um and classification according the diameter. (**B,C**) Slices of 1 µm from different areas of the teeth were analyzed to identify the transversal area of the neural structures and blood vessels. (**D,E**) The innervation comprised 40% of the dental pulp volume and the vasculature another 40% (**F,G**). The majority of the neurovascular structures of dental pulp are within the range of 0.1–50 square microns. (**H,J**) The mid-root and coronal areas of the pulp present more neural and vascular structures within the range of 0.1–10 square microns than the apical area (p < 0.05, ANOVA,). The diameter of the neurovascular structures decreases from the apical section towards the coronal section (**I**,**K**) (p < 0.05, ANOVA).

Lastly, we used an image analyzes software (Imaris, Bitplane Inc) to track (Supplementary Fig. 3), measure and compare the neural and vascular structures of the cleared dental pulp using the surface coating plug-in (Fig. 5). Images were sectioned transversally (Fig. 5A) and a region of interest of 10 µm in thickness was selected and analyzed (Fig. 5B,C). The resulting unit is given in voxels. Aiming to convert the units from voxels into square microns, the slices were reduced to 1 µm in thickness and the algorithm of the surface coating plug-in was able to process the conversion (Fig. 5D,E), thus the results were organized as frequency of distribution as a function of the anatomic site in the dental pulp.

The apical neural bundles have an average diameter of 25 microns (range 2–35 microns), and collectively occupy near 40% of the dental pulp volume (Fig. 5F,H,J). The average diameter of the nerve bundles in the mid-root and coronal areas was 12 and 7 microns, respectively (Fig. 5J). The single nerve fibers measured 0.5–5 µm. Our results are consistent with earlier electron microscopy studies^12,39^ showing that the dental pulp is comprised of small to medium neural structures with transversal areas ranging mostly from 0.1 to 20 square microns (Fig. 5H). Of note, the premolar furcation contained nerve bundles and fibers of a wider range of thickness: 40–60 µm (large), 10–39 µm (medium), and 0.5–5 µm (single fibers).

The average diameter of vessels that entered through the apical foramen was 17 microns and the vasculature occupied another 40% of the dental pulp total volume (Fig. 5C,E,G). According to electron microscopy studies, the blood vessels in human dental pulp can be classified as arterioles (10–50 µm), capillaries (6–10 µm) and venules (10–40 µm)^39^. The diameters of the core blood vessels of the cleared dental pulps ranged from 2 to 30 microns (Fig. 5I,K), which is slightly smaller than the measurements previously described in the literature^42^. This may be due to the evaluation of tissues using different fixation and processing techniques. Regarding the potential tissue distortion caused by CLARITY, the final shape of the samples is a function structural rigidity and porosity of the hydrogel, which is eventually determined by the concentrations of acrylamide, bis-acrylamide, formaldehyde and thermoinitiator. Even though the mixture of 4% acrylamide and 0.05% bis-acrylamide used to process the dental pulps has been described to preserve the tissue structure with minimum distortion^23^, the possibility of shrinkage or swelling of the tissue/polymer cannot be disregarded. Therefore, the measurements show in this study can be at some extent comparable to electron microscopy, however, only a more comprehensive study, including degradation and swelling rates of the samples, a higher number of dental pulps, and algorithm optimization would enable the full comparison between the two techniques.

The software could identify neural bundles, nerve fibers and vascular structures based on the maximum intensity projection. Approximately 500 neural structures enter the apical foramen according to the parameters used by the software (Fig. 5H). Early reports using electron microscopy stated that a human premolar has upwards of 400 myelinated axons shortly after eruption and this number gradually increases to upwards of 700 axons during adult life^36^. Studies by Nair *et al*.^43–45^, using light and electron microscopy showed that healthy human premolars receive an average of 312 (+− 149) myelinated nerve fibers and 2000 non-myelinated axons (range 534–3912) at the juxta-apical pulp. Taken together these numbers are significantly higher than the values found in our work, and a possible explanation is the fact that the CLARITY/Light sheet microscopy method with the PGP9.5 antibody was not always able to distinguish fibers from larger fiber bundles. Also, we found that for both uni- and multiradicular teeth, the thickness of the neural bundles in the core of the pulp decreased towards the coronal part of the pulp^11,12^ (Fig. 5J,K), as classically stated. These results corroborate the use of cleared tissue to perform not only qualitatively investigation of the dental pulp, but also quantitative studies that can are comparable to electron microscopy quantification, with the advantage of being compatible with 3D analyses, of the whole tooth structure.

Additionally, we observed that the frequency of distribution of the blood vessels in the capillary size range, with a transversal section of 0.5–10 microns squares, was twice as high as that of nerve fibers, showing the dense microvascular network comprising the dental pulp (Fig. 5H,I).

The dental pulp innervation includes afferent neurons which conduct sensory impulses as well as efferent sympathetic fibers which provide neurogenic modulation of pulpal blood flow. Sensory fibers of the pulp consist of myelinated A fibers (A-beta and A-delta) and unmyelinated C fibers, both of which are derived from trigeminal nerve and classified according the diameter and conduction speed^1,44,46^. It has been shown that, within the dental pulp, PGP9.5 binds exclusively to neuronal fibers and co-localizes with neurofilament heavy (NFH), a putative myelinated fiber marker^47^. Interestingly, it has also been demonstrated that most fibers within the dental pulp are myelinated and a large subpopulation of these fibers lose their myelin sheath as they approach the subodontoblastic layer and the dentinal tubules. Hence, the uniqueness of the dental pulp innervation merits further exploration using a multitude of markers, since besides its density, demonstrates variations in myelination stages within fibers. The CLARITY technique presented in this manuscript will allow for unprecedented 3D imaging showing the relationship of these different fibers, their variations in marker expression in relationship with other structures of the pulp-dentin complex, and if there is a difference among the type of fibers according to the anatomic location in the dental pulp (periphery versus core and root versus coronal part).

One limitation of this study is the disruption of the odontoblast layer when the dental pulp is removed from the dentin. CLARITY can be used in decalcified tissues^25^ and a recently described PEGASOS technique (polyethylene glycol (PEG)-associated solvent system) was used to clear a whole adult mouse, allowing to image craniofacial tissues, including the dental pulp vasculature^48^, which is far smaller and easier to characterize than the human tooth. Therefore, our goal in this study was to characterize the neurovascular architecture of human dental pulp. Studies in our laboratories are being conducted to develop methods that preserve the pulp dentin interface.

## Conclusion

We present a powerful alternative to gain optical access to the human dental pulp and study the neurovascular architecture of the whole tooth both qualitatively and quantitatively. Dental pulp samples that were CLARITY-cleared and imaged with 3D high-resolution microscopy revealed morphological interactions of the neurovascular system, demonstrating CLARITY to be an outstanding tool to study the structural intricacies of dental tissues in the context of disease and treatment.

## Materials and Methods

### Dental pulp processing for CLARITY

Four healthy human fully formed teeth (premolars and canines) from patients with an age range between 21 and 35 years-old, extracted for orthodontic reasons were utilized. All methods were performed in accordance to the institutional guidelines, and were approved by the regulations set by the Institutional Review Board of the Oregon Health & Science University. Teeth were obtained after informed patient consent from all subjects and collected immediately after surgery. Immediately after extraction teeth were immersed in buffered phosphate saline (PBS) (Sigma, St Louis, MO) with 1% (v/v) penicillin-streptomycin (Sigma), then dissected along the longitudinal axis with a low-speed diamond saw to expose the pulp. Only dental pulps that were retrieved from the pulp chamber whole were used (n = 4).

Samples were immersed in ice cold prepolymer solution made of a mixture of 4% paraformaldehyde (PFA - Sigma), 4% acrylamide (Bio-Rad, Hercules, CA), 0.05% bis-acrylamide (Bio-Rad), 0.25% VA-044 thermal initiator (Wako Chemicals USA, Inc) in PBS and were left fixing at 4 °C for 72 hours. After that, to initiate polymerization, the conical tubes with the dental pulp and hydrogel prepolymer were placed in a vacuum chamber and degassed for 10 minutes, next the chamber was flooded with pure nitrogen gas to displace oxygen and to ensure consistent hydrogel polymerization. The vacuum chamber was placed in an incubator at 37 °C for 3 h. Once the hydrogel was polymerized, excess gel was removed from the pulp tissue by wiping the surface with lint-free tissue paper (KIMTECH).

Samples were then placed into clearing solution consisting of 200 mM boric acid (Sigma), 4% (w/v) sodium dodecyl sulphate (SDS) (Sigma) dissolved in deionized water with addition of NaOH (Sigma) to reach pH 8.5. Clearing solution was replaced after 48 h to remove residual hydrogel and then replaced once a week for 8 weeks at 37 °C as stated for passive clearing^23^. After achieving near optical transparency (Fig. 1), the pulps were washed in PBST (1x PBS with 0.1% Triton X_100) (Sigma) for 24 hours then immunostained.

### Immunofluorescence stain

Dental pulp-polymer hybrids were rinsed with PBST (0. 1% Triton X-100 (Sigma) in PBS) for 24 h. For endothelial stain samples were incubated with primary mouse monoclonal antibody anti-CD31 (clone 89C2, # 3528, Cell Signaling), 1:100 in PBST and for neural stain incubation was with primary rabbit polyclonal anti-PGP9.5 (Accurate Chemical and Scientific Corp., Westbury, NY) or UCHL1/PGP 9.5 (rabbit polyclonal, Proteintech cat # 14730 1-AP) for 48 h at 37 °C. After 48 hours we replenished each primary antibody and continued incubation for an additional 48 h at 37 °C to ensure uniform antibody distribution throughout the entirety of the tissue. After the primary antibody incubation samples were rinsed with PBST for 24 h at 37 °C and incubated with the secondary antibodies for 96 h at 37 °C, with an antibody replenishment at 48 h. For the primary anti-CD31 the secondary antibody used was donkey-anti-mouse IgG, A10037 (Alexa Fluor 568, Life Technologies) and for anti-PGP9.5 the secondary was goat-anti-rabbit IgG, A11034 (Alexa Fluor 488, Life Technologies) (dilution 1:100).

After PBST rinses, the samples were transferred into 85% glycerol for 24 h at 25 °C to achieve a refractive index matching to 1.45 refractive index (RI) and minimize light scattering. Next, the samples were mounted in conic plugs of 1% low temperature melting agarose (A4018, Sigma-Aldrich), which were allowed to set inside 1 cc syringes. Lastly, the agarose plugs with the samples inside were suspended into 85% glycerol solution for RI = 1.35, or PROTOS for RI = 1.46 [23.5% (w/v) *n*-methil-*d*-glucamine, 29.4% (w/v),diatrizoic acid, 32.4% (w/v) iodixanot]^49^, matching of the agarose and dental pulp together.

### Digital imaging and quantification

Whole dental pulp samples were imaged with a Zeiss Lightsheet Z.1 microscope (Zeiss, Germany) equipped with detection objectives of LD-Plan-Apochromat 5 × 1.0, nd-1.33. Imaging was performed using one or dual side illumination, depending on the size of the sample. For PGP9.5 imaging the excitation used was at 488 with an emission of 520 and for CD31 Alexa fluor-568 imaging the excitation used was at 577  with an emission of 572 nm. Some samples (Fig. 4) and Supplementary Fig. 2B,C) were also imaged with confocal microscope (Zeiss, LSM 880, Germany) with objective of 20 × (Zeiss, Plan-Apochromat 20x/0.8 M-27).

Images were fused using Imaris Stitcher 9.2.0 (v9.1, Bitplane – Oxford Instruments, Zurich, Switzerland) (with an overlap of 10% to 15%) or were converted into Arivis Vision 4D format (Germany). Tiles were stitched creating large field of view stacks. Data sets were visualized as projections and 3D volumes.

We traced the 3D course of a nerve bundle activating the measurement point of the Imaris software, which is a multidimensional analysis program based on fluorescence intensity data was used for vessel and neural structures quantification. We chose a nerve bundle of interest and manually traced the path of the structure considering X-Y-Z directions using the plug-in “Filament” and skipping the automatic filament tracer given the complexity and length of the nerve fiber structure.

For the measurement the neural or vessel structures surface the area module of Imaris was used. The image was sectioned transversally using the ortho slicer tool, and a region of interest of 10 µm in thickness was selected, the module of surface creation was then activated, and one channel (red or green) was analyzed (Fig. 5B,C). We used the thresholding of absolute intensity to identify the total neural or vessel structures in the region of interest and later using slices of 1 µm the statistics coded color set up was activated to transform the surface area of the transversal section of the neural structures or vessels into square microns (Fig. 5D,E). Also the diameter size of the nerves and vessels in microns was measured using the Imaris slice view. The data was compiled and presented as frequency of distribution of the vessels/nerves statistically analyzed using one-way ANOVA with Tukey as a post-hoc test (Graphpad Prism 7, San Diego, CA, USA). The accepted alpha was of 0.05.

To remove edges and background from some images used for illustration purposes (Fig. 1A and Supplementary Fig. 1), we opened the files with Imaris, cropped the desired region of interest and exported the images as.Tiff series and separated the Tiff series from each channel into individual folders. Each folder was loaded into ImageJ (Free Software, NIH, Bethesda, Maryland, USA) as an Image Sequence, later the plugin BaSic was activated and processed with the default settings (choose to estimate both flatfield and darkfield corrections). The corrected images were saved into a single folder and reloaded into Imaris. We used original files to input correct voxel settings.

## Supplementary information

Supplementary movie 1

Supplementary movie 2

Supplementary movie 3

Supplementary movie 4

Supplementary movie 5

Supplementary movie 6

Supplementary movie 7

Supplementary movie 8

Supplementary figures

## Data Availability

Data available on request from the authors.
