# Supplementary material for: 3D-Imaging of Whole Neuronal and Vascular Networks of the Human Dental Pulp via CLARITY and Light Sheet Microscopy
Source: Sci Rep. 2019 Jul 26;9:10860. doi: 10.1038/s41598-019-47221-5 (PMC6659648; doi:10.1038/s41598-019-47221-5)
Supplement: Supplementary file 9 — Supplementary figures [file 41598_2019_47221_MOESM9_ESM.pdf]

3D-Imaging of Whole Neuronal and Vascular Networks of the Human Dental Pulp via CLARITY and Light Sheet Microscopy

Cristiane Miranda França[1], Rachelle Riggers[2*], John L. Muschler[2], Matthias Widbiller[3],* Peter Manning Lococo[4], *Anibal Diogenes[4], and Luiz Eduardo Bertassoni[1,5,6,7]*

Supplementary figure 1 - Vascularization and innervation of a human canine tooth

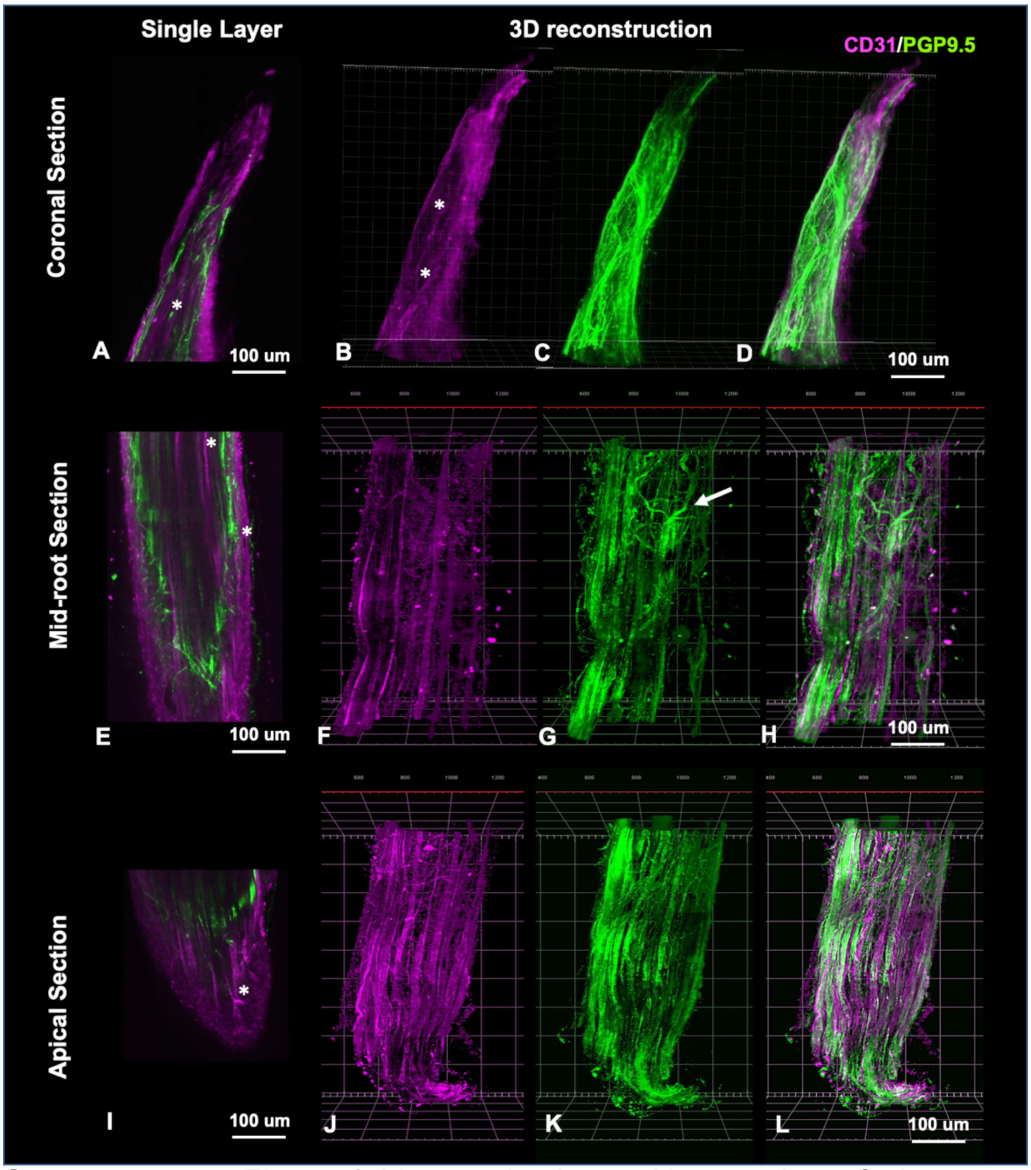

Supplementary Figure 1: Vascularization and innervation of a human canine tooth showing tiles from the apical, mid-root and coronal sections. A comparison is done with the slices in 2D (A,E,I) and the rendered image in 3D. The neurovascular structures present a predominantly straight course in the apical site (J,K,L), at the mid-root level the splitting and branching of the structures becomes more evident (F,G,H and arrow). At the apical level medium-sized core vessels and neural bundles can be observed (B,C,D). Coronal dental pulp presents core vessels with diameters of 14-10 microns (*). The capillary network in the periphery causes an increase of the background signal (A,B).

Supplementary figure 2 - Vascularization of the dental pulp

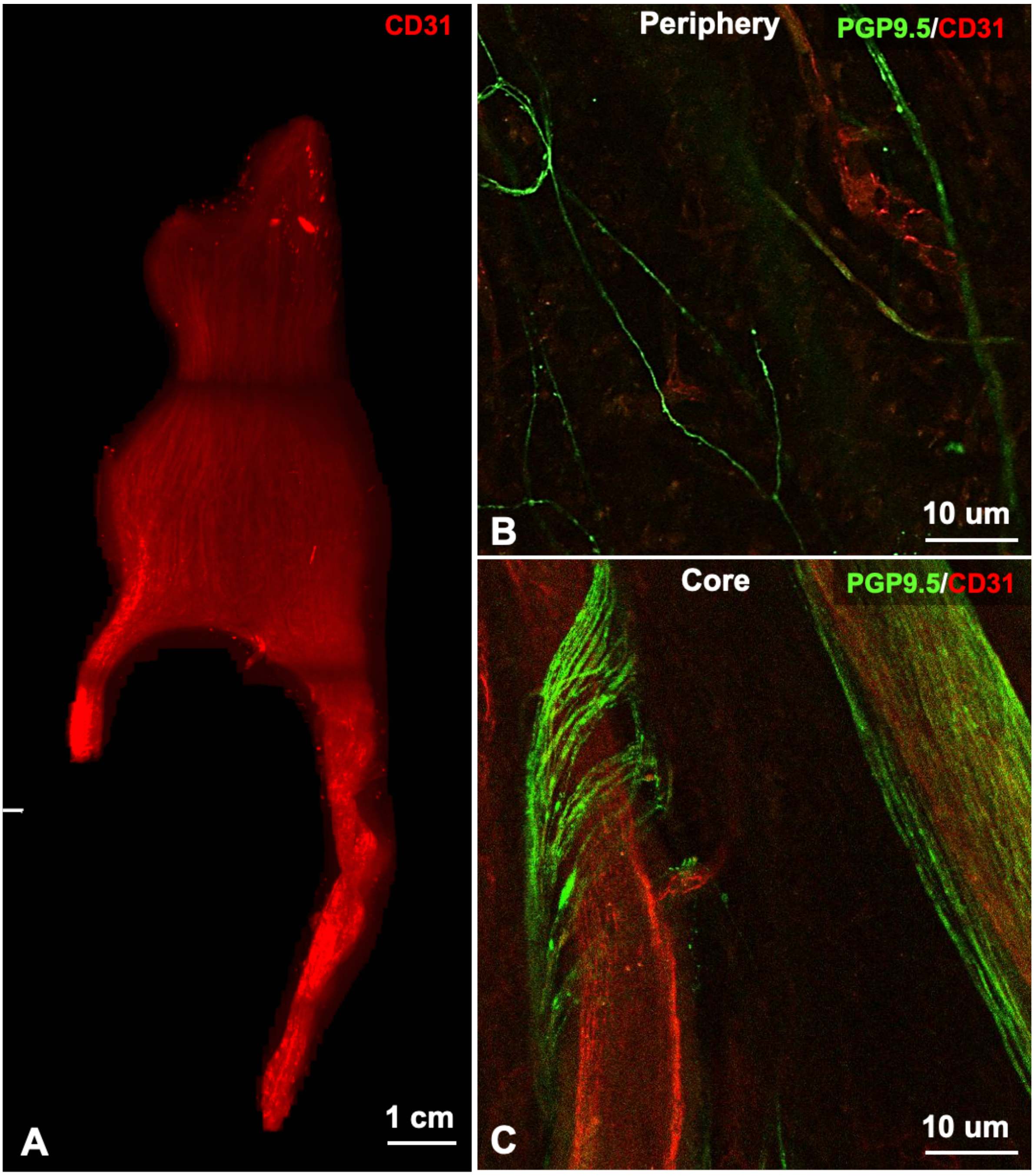

Supplementary Figure 2: Vascularization of a premolar (A) stained for CD31. (B) detail of the innervation and vasculature interplay in the periphery of the premolar. (C) Nerve bundle surrounding a vessel in the core of the tooth (red – CD31, green – PGP9.5)

Supplementary Figure 3 – Different interplay of neuro-vascular structures of the dental pulp

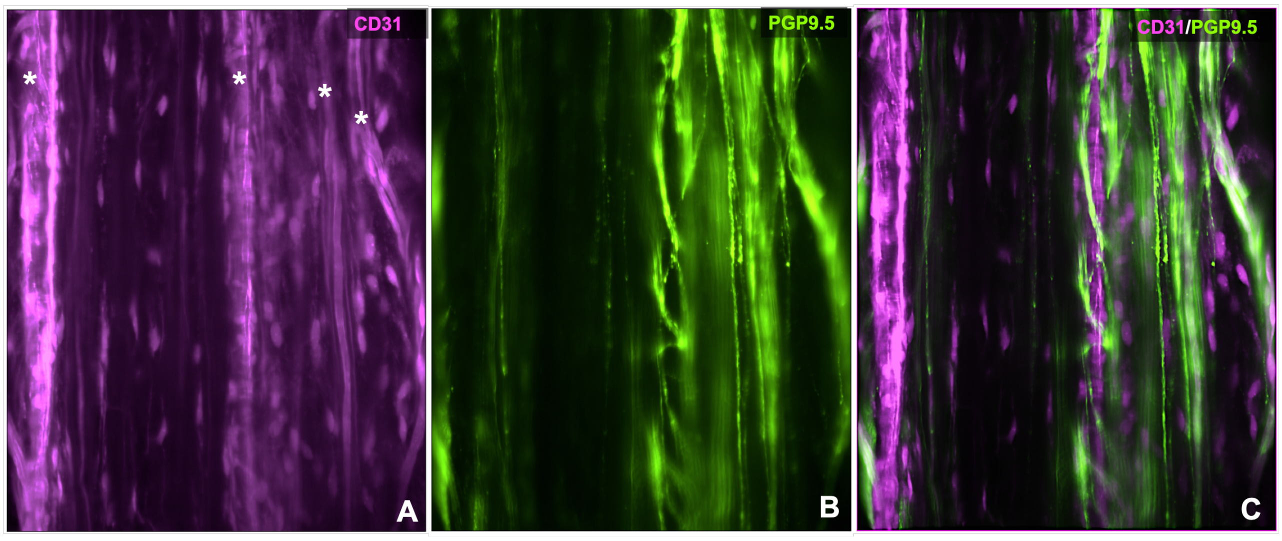

Supplementary figure 3 - High resolution images of neural and vessels showing different arrangements. From left to right - arterioles (asterisks) can be positioned longitudinally with nerves, or nerves can partially surround arterioles in spiral, or nerves can surround completely the vessels.

Supplementary figure 4 – Neural Tracking

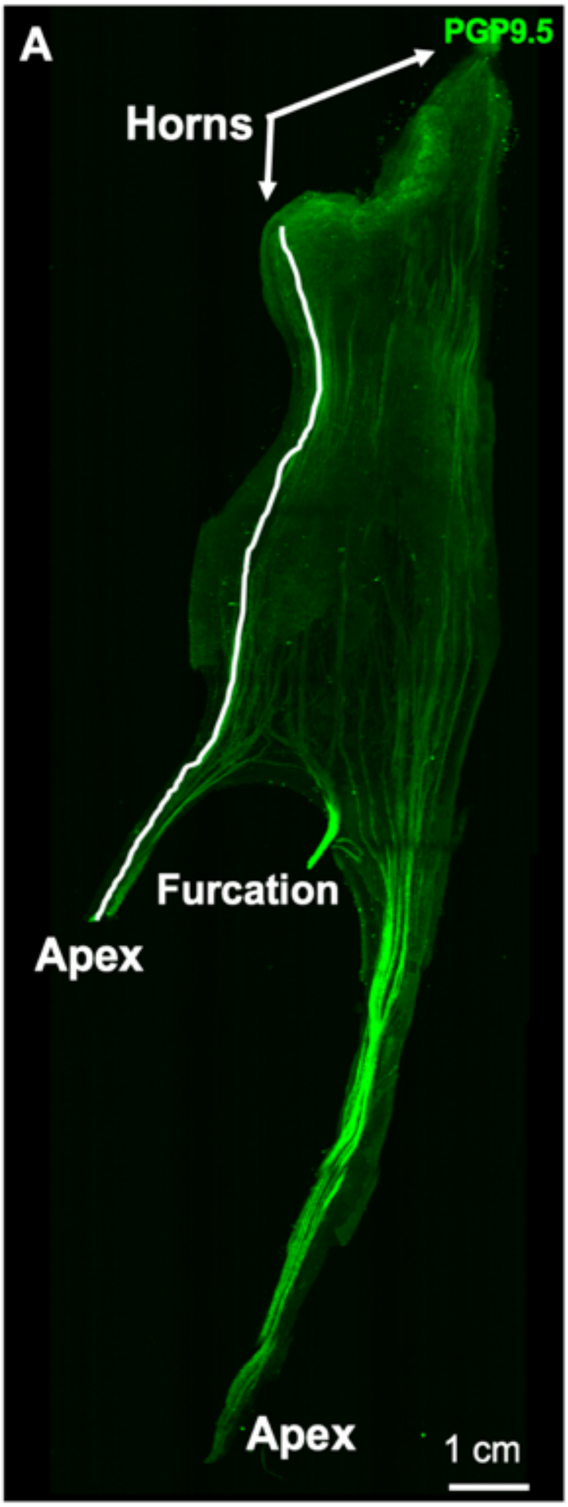

Supplementary figure 4 – Tracking the path of a nerve bundle of a premolar tooth from apex to crown.
